# Strengthening Relationships Within Intensive Psychiatric Care: Staff Perceptions of an Attachment Training Intervention

**DOI:** 10.1111/jpm.13109

**Published:** 2024-09-22

**Authors:** Aiden Duffy, Karen Goodall, David Carmichael, Sean Harper, Katy Sivyer, Kathy Carnelley, Tess Maguire, Katherine Newman‐Taylor

**Affiliations:** ^1^ University of Edinburgh Edinburgh UK; ^2^ NHS Lothian Edinburgh UK; ^3^ NHS Grampian Aberdeen UK; ^4^ University of Southampton Southampton UK

**Keywords:** attachment, inpatient, psychosis, qualitative, staff

## Abstract

**Introduction:**

Intensive psychiatric care units can be challenging environments in which to build strong staff‐patient relationships. Attachment theory may provide a useful model for strengthening relationships in this setting.

**Aim:**

The aim of this study was to explore staff perceptions of the utility of attachment theory for understanding patient behaviour in the intensive psychiatric care setting.

**Methods:**

Semi‐structured interviews were conducted with 11 multi‐disciplinary staff members. Interviews focused on the staff member's experience of learning about attachment theory and applying this perspective to their work on the ward. Transcripts were analysed using reflexive thematic analysis.

**Results:**

The analysis led to the development of three themes: *engaging the reflective self*, *new perspective on others* and *cohesive ward culture*.

**Conclusion:**

Staff reported that learning about attachment theory supported them to better understand patient distress and associated behaviours. Notably, staff also used attachment theory to reflect on both their own internal states and the internal states of their colleagues. These reflections were suggested to bolster staff wellbeing and improve the ward milieu.

**Implications for Practice:**

Integrating attachment theory into team formulation, ward rounds and reflective practice groups could have a benefit for clinical practice, staff wellbeing and team cohesion in this setting.

**Summary**

- Relationships are central to recovery in mental illness. However, acute psychiatric wards are notoriously difficult environments in which to build relationships.
- Attachment theory provides a framework that can be used to strengthen relationships in this setting.
- We taught staff working on an acute psychiatric ward about attachment theory and helped them to apply this knowledge to their work on the ward. We then asked staff how their new knowledge had changed how they developed relationships on the ward.
- Staff told us that being aware of attachment theory helped them to better understand their patients and their colleagues. It also improved the overall atmosphere on the ward.

## 1 Introduction

The importance of the therapeutic relationship is well recognised in the treatment of mental health difficulties (Baier, Kline, and Feeny 2020). Previous studies have found that patients perceive their relationships with staff as central to their recovery (Walsh and Boyle 2009; Wood et al. 2019). However, some settings, such as acute psychiatric wards, can be challenging environments in which to build relationships (Bolsinger et al. 2020). Furthermore, some mental health symptoms, for example, the paranoia that can occur in people experiencing psychosis, can disrupt the attempts of professionals to form relationships with their patients (Pipkin, Hogg, and Armitage 2021). A systematic review conducted by Bucci et al. (2015) concluded that attachment theory may provide a useful framework for informing and improving the relational dynamics of mental healthcare settings. Although the review was narrative, and did not synthesise outcome data, it provided a theoretical argument that attachment theory could add value in the assessment, formulation, treatment and discharge phases of intervention (Bucci et al. 2015).

### 1.1 Attachment Theory

Attachment theory posits that the sensitivity, responsiveness and availability of our primary caregivers in early life influence how we respond to distress across the lifespan (Bretherton and Munholland 2018). This process is hypothesised to occur through the development and maintenance of Internal Working Models (IWMs) (Bowlby 1969). These cognitive‐affective structures, which incorporate beliefs and expectations about both ourselves and others, are suggested to guide our care‐seeking behaviour at times of distress (Bretherton 2013). People whose IWM imbues a sense of self‐worth, a confidence in their ability to cope with reasonable threats and an expectation that others are supportive are described as experiencing attachment security (Gillath and Karantzas 2019). Those who have less positive beliefs and expectations about themselves and others are described as experiencing attachment insecurity (Kobak and Bosmans 2019).

In adulthood, attachment insecurity is commonly measured on two dimensions: attachment anxiety and attachment avoidance (Brennan, Clark, and Shaver 1998). People who are high in attachment anxiety have a low expectation that they can cope with threat. They predict that others will be inconsistent in the care they provide, and, as such, people with attachment anxiety learn that amplifying their emotions may increase the likelihood of getting a response from caregivers (Clark et al. 2020). People who are high in attachment avoidance have experienced consistently unavailable caregivers and, as such, have learned that they must cope with threats without the support of others. When faced with difficult situations they will downregulate their distress to demonstrate their own independence and subsequently dismiss offers of support from other people (Mikulincer and Shaver 2019). There is an overrepresentation of attachment insecurity in people who experience severe mental health difficulties, such as psychosis and bipolar disorder, compared with the general population (Carr, Hardy, and Fornells‐Ambrojo 2018; Herstell et al. 2021). The association between attachment insecurity and severe mental health difficulties is of particular relevance when taking into account the treatment pathway for those experiencing acute psychological distress.

### 1.2 Attachment Theory and Acute Psychiatric Care

People experiencing acute psychological distress may require inpatient care when their difficulties present a risk to themselves or others. Those whose risk cannot be safely managed in an open acute inpatient ward may be transferred to a more secure ward, known as an Intensive Psychiatric Care Unit (IPCU). Whilst people who have experienced acute inpatient psychiatric care report that they can find the ward environment threatening, they also identify that the development of meaningful relationships with ward staff can provide a sense of safety that can support their recovery (De Ruysscher et al. 2020). However, patients often perceive staff to be too busy with non‐clinical responsibilities and large caseloads to find time to develop relationships (Hopkins, Loeb, and Fick 2009; Staniszewska et al. 2019). Qualitative research with staff has also found that despite being motivated to connect with their patients, staff perceive there to be multiple barriers in forming relationships with patients in this setting (Wood et al. 2019). These include fluctuating levels of wellness within patients and limited access to training in psychological approaches (Boniwell et al. 2015; Wood et al. 2019).

Given the relational focus of attachment theory, the role of attachment in acute inpatient psychiatric care is increasingly being investigated (Berry 2021). For example, a recent small‐scale cross‐sectional study found that the attachment orientation of both patients and nurses is correlated with their ability to regulate their emotions and manage distress in an acute inpatient mental health setting (Wainwright et al. 2021). Studies that assess the impact of a patient's attachment on their engagement with acute inpatient mental health services are difficult to synthesise due to the diversity of attachment measures that are used across studies (McGonagle et al. 2021). That considered, the available data indicates that if attachment remains unacknowledged in this setting, there is a risk that staff could misinterpret the behaviour of patients leading to patients being viewed as disengaged or demanding with negative consequences for staff‐patient alliance (Bucci et al. 2019).

**FIGURE 1:**
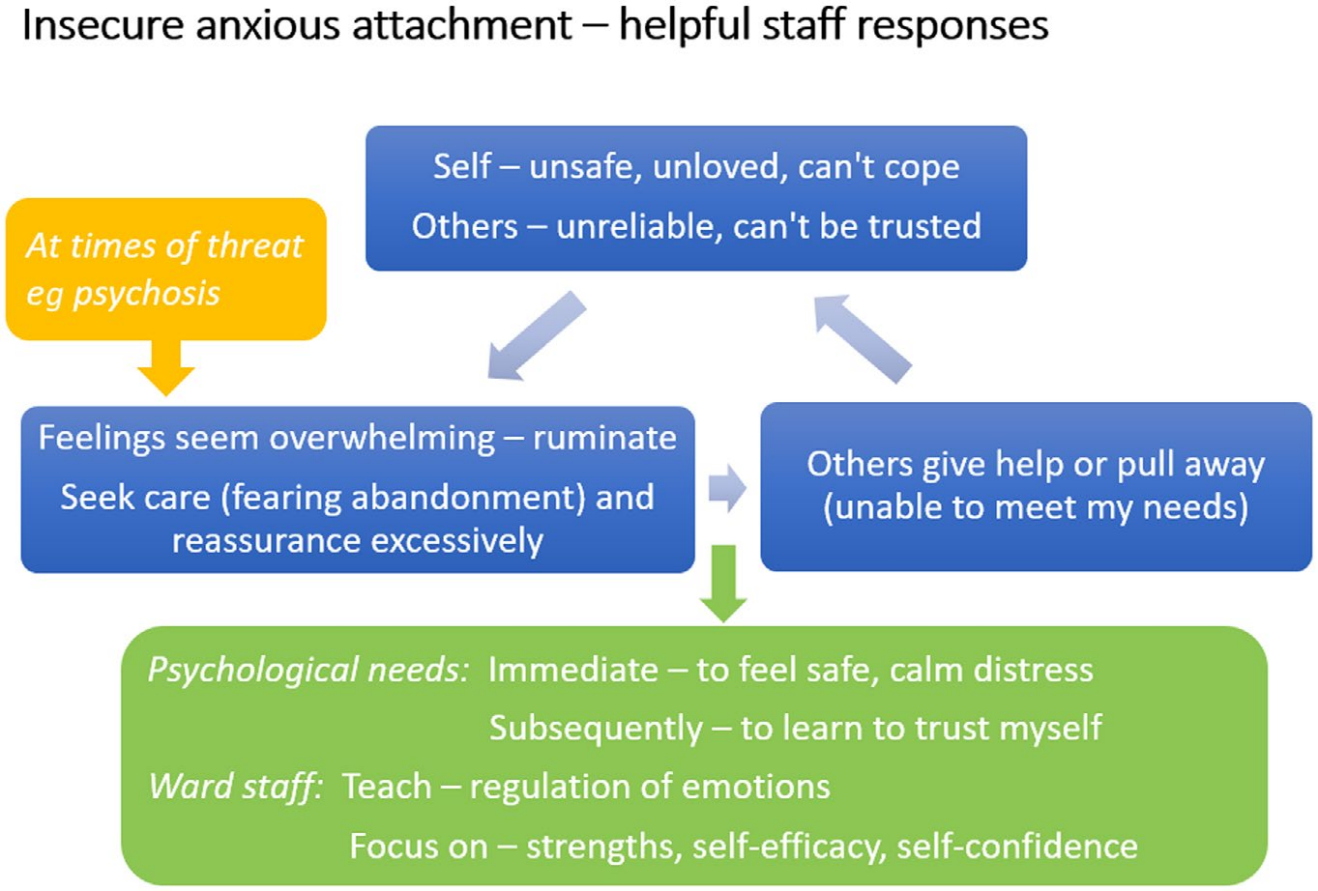
Example of Newman‐Taylor et al.'s (2022) attachment‐based cognitive‐behavioural model (anxious attachment).

Until recently, there has been no psychologically informed strategy to support staff in adapting their approach to developing therapeutic relationships with patients based on their attachment style (Bucci et al. 2015). Addressing this gap, Newman‐Taylor et al. (2022) have developed attachment‐based cognitive‐behavioural models for psychosis. Figure 1 demonstrates a cycle for a person with an anxious attachment style. Packaged as part of a wider attachment‐focused staff training programme referred to as ‘Safe & Secure’, these models offer a new tool to aid staff in developing a shared understanding of the attachment needs of those admitted to acute inpatient psychiatric care. However, the utility of Newman‐Taylor et al.'s (2022) models, and their influence on staff attitudes and behaviours, has yet to be assessed in a clinical setting.

### 1.3 Current Study

There has been a long‐standing indication within theoretical literature that an attachment‐informed approach may have utility for strengthening staff‐patient relationships within inpatient settings (Bucci et al. 2015). However, clinical research to support this theory has not yet been developed, nor have tangible strategies for implementing an attachment‐informed approach within an inpatient setting. The recent development of attachment‐informed cognitive‐behavioural models for psychosis (Newman‐Taylor et al. 2022) presents an opportunity to address this gap in the literature. The present study sought to understand the experience of IPCU staff when learning about and applying the attachment‐informed approach to patients who experience severe mental health conditions. The aim of the study was to explore staff perceptions of the utility of attachment theory for understanding patient behaviour and strengthening staff‐patient relationships within an IPCU.

## 2 Methods

### 2.1 Participants

Staff were recruited from a mixed‐sex, ten‐bed adult IPCU located within a large general hospital in Scotland.

Participants were eligible for inclusion in the study if they (i) were over 18 years of age; (ii) were a permanent member of the ward team and had worked on the IPCU for more than 1 month; (iii) had daily clinical contact with patients experiencing acute psychological distress; and (iv) had attended the Safe & Secure workshop and been working on the ward during the 8‐week integration phase.

### 2.2 Safe & Secure Training

Safe & Secure is an 8‐week staff training programme designed for staff working in inpatient settings. The programme aims to improve staff knowledge of attachment theory and the links between attachment insecurity and a range of severe mental health conditions. The programme consists of a 2‐h workshop in which participants are provided with an overview of the key concepts of attachment theory before being introduced to Newman‐Taylor et al.'s (2022) attachment‐based cognitive‐behavioural models. Trainees then discuss how these models apply to patients on the ward. This is followed by an 8‐week integration period. The integration period includes the introduction of attachment theory into wards rounds, team formulations, reflective practice groups and case consultations. Additional information on the content of the training programme is available in Appendix A.

The 2‐h workshops were delivered by the ward Clinical Psychologist (DC) with support from the lead researcher (AD). Four workshop sessions were held across the course of a 5‐week period to ensure as many staff as possible were able to access the training. The ward Clinical Psychologist (DC) was solely responsible for implementing the training during the integration phase. All training was delivered face‐to‐face.

### 2.3 Procedure

Following the delivery of the Safe & Secure workshops, a written overview of the study, with details of the lead researcher, was emailed to all ward staff. Those who contacted the lead researcher (AD) were sent participant information sheets and consent forms before being recruited into the study.

Semi‐structured interviews lasting on average 41 min (range 32–52 min) were conducted by the lead researcher (AD). Interviews were conducted over a 3‐month period with the first interview occurring 3 months after the last training session. The majority of interviews took place within 4 months of the final training session (*n* = 9); however, due to increased acuity on the ward during the data collection phase, two participants were interviewed almost 6 months after the training session. Interviews took place online (*n* = 8), or in‐person in a private space within the IPCU (*n* = 3). A debrief form was provided on study completion. Digital recordings of the interviews were deleted following verbatim transcription.

The interview schedule was developed by AD, KG and KS. It focused on the experience of learning about, and applying, the attachment‐focused approach and included questions such as ‘What did you feel when learning about attachment theory?’, ‘What difference, if any, has the training made to how you form relationships with patients?’.

#### 2.3.1 Ethics Statement

Ethical approval for the study was provided by the University of Edinburgh's Research Ethics Committee. The research was conducted in agreement with the Department of Clinical Psychology, NHS Lothian. The project was deemed a service evaluation by the local NHS ethics panel and, as such, all management of the project remained within the research team.

### 2.4 Analysis

#### 2.4.1 Qualitative Analysis

Given the proximity of the research team to both the intervention and the research setting, reflexive thematic analysis (TA) was selected as the method of data analysis. The analytic process adopted a critical realist epistemology and was framed within an experiential paradigm. This paradigm prioritised participants' meaning‐making as they reflected on the changes that the training brought to their own values and clinical practice. Due to the paucity of previous related research, the data analysis process was inductive. The focus of the study on both the experience of participating in the training programme and on the lived experience of working on the ward directed the analytic process to seek meaning at both a semantic and a latent level. As is standard practice in reflexive TA, all data analysis was conducted by the lead researcher (white, male, mid‐30s) (AD). The analytic process, described in Table 1, followed the six steps of thematic analysis (Braun and Clarke 2006).

**TABLE 1:** Stages of thematic analysis.

Stage	Process
Familiarisation	The lead researcher spent time becoming familiar with the data by re‐reading the transcripts
Generating initial codes	Two rounds of coding took place. Codes were written beside each transcript before being collated in an electronic document. Code titles were standardised across scripts
Searching for themes	Codes that occurred across transcripts were grouped by concept to form subthemes. Unused codes were then re‐assessed to see if they fitted within a theme or were to be discarded. The discarded codes mainly related to the logistics of implementing the programme and did not attend to the change that the programme brought about. The analyst's initial groupings divided codes into those that related to the participant's internal experience of the programme and how the programme changed their external behaviour
Reviewing themes	Transcripts were reviewed to ensure that the themes accurately represented the ideas that participants were sharing. During this stage, the analyst began to re‐assess their initial division of codes into two groupings (internal/external) and began to develop a third theme that related to the systemic influence of the programme. This theme was considered distinct given its focus on the shared experience and influence of the training, as opposed to individual‐level changes
Naming themes	Theme titles that provided sufficient clarity without losing the core concept of the grouped codes were drafted. Given the focus of the programme on influencing the perspectives and behaviours of staff, theme titles were selected that best represented the change mechanisms being discussed by staff. Theme titles were further elucidated through reflective conversation with KG
Producing the report	The report was written with acknowledgement of the recommendations for quality practice in thematic analysis (Braun and Clarke 2023)

### 2.5 Reflexivity Statement

Several of the individuals who were directly responsible for the development of the Safe & Secure programme were consulted during the development of this project. Due to this, the lead researcher (AD) was conscious of an inner sense of wanting to ‘get things right’ within the research process to avoid biasing the study process. AD recognised that this feeling led to internal tension during the administration of the initial interviews. However, AD felt that his lack of previous experience in the IPCU setting was helpful during the analysis phase. As an analyst, AD held no prior expectations for how care in an IPCU ‘should look’ thus supporting the inductive approach to analysis.

## 3 Results

A total of 20 staff members participated in the Safe & Secure programme (80% of ward staff). The integration phase comprised: case consultations (*n* = 4), ward rounds (*n* = 4), reflective practice sessions (*n* = 4), team formulations (*n* = 3). The integration phase required 20 h of input from the ward Clinical Psychologist. At the point of data collection, 16 of the staff members who had attended the programme continued to work on the ward. A total of 11 (68.75%) of the available staff members participated in the study. The majority of participants were female (72.7%) with a mean age of 38.4 years (range 24–61). The average length of NHS employment was 8.27 years (range 1–30). Participants came from a range of disciplines including nursing, art therapy, psychiatry, activities co‐ordination, clinical support work and occupational therapy.

### 3.1 Thematic Analysis of Staff Experience

Based on the grouping of related codes from across the dataset, three themes were developed. An overview of the themes can be found in Table 2.

**TABLE 2:** Themes developed through reflexive thematic analysis.

Theme title	Summary	Subthemes
Engaging the reflective self	The changes in perspective that occurred within staff as a result of reflecting on the role of attachment in both their personal and professional lives	Value of recognising own attachment style Detaching from immediate reactions Creating space to reflect
A new perspective on others	The changes in the way that staff perceived the behaviour of patients and colleagues after the training	It's not personal Increased empathy Biographical contextualisation Reframing ward interactions
Cohesive ward culture	How the culture of the ward changed as a result of the training programme	Shared knowledge/language Recognising individual differences across the team Increased confidence in team

#### 3.1.1 Engaging the Reflective Self

The first theme related to the self‐reflection that occurred for participants as they learned about attachment theory and considered how attachment had influenced their personal and professional lives.

##### 3.1.1.1 Value of Recognising Own Attachment Style

The training prompted staff to reflect on their own attachment style and how this interacts with their patient's attachment style. Having insight into your own attachment style was reported to bring a deeper understanding of the personal challenges of inpatient work.

(thinking about) one patient in particular, how his attachment style was then creating a reaction for my own attachment style… if I understand my own attachment style, then I'll understand what's being triggered within me. (P3)

For many staff, being more aware of their own self‐regulation strategies, and noticing when these strategies were activated, facilitated self‐reflection.

When I was in challenging scenarios… and where I was feeling stressed, I then figured out what it was about that was making me feel that way. (P1)

##### 3.1.1.2 Detaching From Immediate Reactions

There was a sense that staff often spent time thinking about their patients when they were away from the IPCU. These thoughts were often self‐critical with staff spending more time thinking about their perceived failures.

(when patients don't engage) it can make you feel, yeah, bad at your job. (P5)

Self‐awareness, and an ability to reflect on one's own internal world, was suggested to be helpful in challenging some of this ruminative thinking. For some staff, an increased insight into their own attachment style was reported to lead to less self‐criticism and therefore increased wellbeing.

And it makes me feel less stressed and less burned out because there's always a reason and like it's not actually even really my fault that I feel this way because of XYZ. (P8)

Participants reflected that the benefit of the programme came from an active engagement with the materials and the opportunity to reflect on attachment theory.

It gave you chance to think because sometimes… you are on auto‐pilot. You just do things and you're not really thinking about like attachment. I would never have thought that what I do would fall under the category attachment. (P15)

In spite of the perceived value of active engagement with the training programme, many participants found it difficult to make time to attend the team formulations and reflective practice sessions that occurred during the integration period.

##### 3.1.1.3 Creating Space to Reflect

Participants viewed the 2‐h training session as a space in which they could explore their own personal reactions to patients in a way that they had not before. A couple of participants highlighted self‐disclosure by the training facilitators as important to setting the tone for the session.

I think having a clinician speak about attachment is one thing, but having them apply it is another that makes it so much more meaningful. (P4)

The value of reflection was consistently highlighted by nearly all participants. In the quote below, P7 frames reflection as a chance to ‘step back’.

So just being able to step back for a second….yeah, just having a bit more… thought about ‘why we're doing it and how we're doing it’. (P7)

Reflection was not considered to come without its challenges. Self‐reflection was considered by some participants to provoke difficult questions about the quality of the care they were providing.

I sit and think ‘what could have done better?’….you see patients coming here and they are really unwell but when you see them leaving and there is such an improvement. So you've obviously done something right. Even if it's hard to put yourself through that. (P15)

#### 3.1.2 A New Perspective on Others

The second theme captured how staff perceptions of other people's behaviour changed once they acknowledged the other person's early life experiences and considered how these experiences may have shaped their IWMs. This shift in perception led to positive changes in staff emotion and behaviour.

##### 3.1.2.1 It Is Not Personal

All participants highlighted the emotional burden of inpatient work and, in particular, the feeling that they were personally responsible for the recovery of patients. Recognising that challenging patient behaviour could be driven by the activation of a patient's attachment‐related expectations, as opposed to due to staff failure, helped staff to relieve some of this sense that they held sole responsibility for the patient's recovery.

It feels like less of a personal attack. If something is part of somebody's pattern of behaviour, then it's not you as a clinician. (P1)

Furthermore, recognising that patient behaviour may be attachment‐related, as opposed to malicious, helped participants to consider that patients may not be fully conscious of the consequences of their behaviour.

These are not things that the patient has control over…they are part of mental illness and the extremes of your attachment style coming out. I think that really helps with viewing them as unwell and needing help rather than as bad. (P6)

However, when the ward environment became particularly chaotic or acute, many participants found their ability to reflect on the psychological drivers of patient behaviour to be limited.

Your mind is really just thinking about survival in that moment, and then you're not really thinking about attachment styles and trying to rationalize what's going on. (P3)

##### 3.1.2.2 Increased Empathy

Reframing patient behaviour as attachment‐driven, as opposed to intentional, allowed staff to feel a greater level of understanding and, ultimately, compassion towards their patients.

The training did allow you to kind of increase your tolerance towards that kind of behaviour …because you had a better understanding of why they were behaving that way. A kind of wider window of tolerance… and more kind of compassion. (P5)

The idea of greater empathy as a mechanism by which staff increased their tolerance to challenging behaviour was present throughout the transcripts. In response to being asked what changed for staff when they felt more empathy for patients, P6 responded:

I think that improves their resilience to… whatever's thrown at them. …. I think that it makes them feel that, you know, despite the day being hard and challenging, it's worth it. (P6)

##### 3.1.2.3 Biographical Contextualisation

Several participants reported that thinking about the difficult experiences that their patients had experienced in early life, and how this was now disrupting their attempts to recover from mental illness, was upsetting.

You could acknowledge then, that some people, because of their early experiences, don't get quite the same treatment…that it can be a bit different. (P9)

Patients who staff perceived to have attachment avoidance were mentioned as a group who staff were now more aware of. Participants reflected that patients who spent more time in their room were historically overlooked. Recognising that this behaviour may be the patient's attempt to self‐regulate led to concerns about inequity of care.

It ends up being some kind of inequity in terms of who and who gets staff attention… because there are some people who are just very avoidant…and it's kind of sad to think that's you know a pattern from their childhood that's still affecting their healthcare. (P1)

##### 3.1.2.4 Reframing Ward Interactions

Participants reframed the behaviour of their colleagues through an attachment lens. Participants reported a greater understanding of their colleagues' behaviour and an increased empathy.

The opportunity to speak with staff about their experience of attachment… helped me grasp better some of what was going on for them…it's so easy to fall into a good guys, bad guys mode of thinking. (P4)

A similar process of reframing occurred in relation to patient behaviour. For example, P13 recognised that identifying their own attachment style during the training session had helped them to recognise that not all people had experienced the same early caregiving environment.

It makes me understand that they didn't have what I had, so, therefore, this can be the reason that behaviour has happened. (P13)

Despite this shift towards considering the role of a patient's attachment‐related expectations in driving their behaviour, when asked which patients they found most challenging to build relationships with, the patient's gender, forensic history and diagnosis were the most frequently mentioned obstacles.

#### 3.1.3 Cohesive Ward Culture

The final theme drew together the comments that participants made about the changes that they felt had occurred at an organisational level due to the implementation of the programme.

##### 3.1.3.1 Shared Language

The training was considered by participants to provide ward staff with a shared language that made it easier to share their clinical perspectives with other team members.

I want to say a sense of relief…this is a common language that we can use. It's not going to be something that I have to explain. (P4)

Participants reported that this shared understanding, combined with the advice provided in the training session, allowed the team to take a cohesive approach to the treatment of individual patients.

It's good to kind of have that shared understanding of why the person is presenting the way they are and how we can approach it all in the kind of same way. (P5)

Whilst participants valued having a shared language, it was noted that two participants demonstrated some confusion when speaking about the attachment categories. One participant, for example, described working with a patient who demonstrated a pattern of behaviour associated with avoidant attachment before labelling this patient as having an anxious attachment style.

##### 3.1.3.2 Recognising Individual Differences Across the Team

Four of the participants held responsibility for supervising other staff. These participants considered how they could use the training to support their supervisees.

It has definitely changed the way that I approached the staff… when they're getting quite anxious on the ward…actually they probably just want to vent about it and they are probably not as anxious as they think they are… it's been a really helpful tool… to kind of promote psychological safety in the ward. (P8)

For many staff, regardless of grade, learning more about attachment theory increased their understanding of the relational dynamics of the ward team. In the quote below, P13 invokes the attachment‐focused approach when reflecting on how having a range of personalities working on the ward can be valuable.

You have different types of staff. So, you have the more vocal one, you have the softener…and now knowing that there's different attachment styles, you can actually make the nurses a better fit for the patient. (P13)

However, many staff cited the high turnover of staff and a reliance on temporary staff as barriers to the continued use of training. P7 explained: ‘staff in bank agencies haven't… necessarily been embedded within the multi‐disciplinary team’ (P7).

##### 3.1.3.3 Increased Confidence in the Team

The training was considered to empower staff members to formulate and understand patient behaviour without requiring the input of the ward Clinical Psychologist.

[when facing difficult situations on the ward]. if it wasn't for that training then I'd maybe speak to the ward psychologist because they know better. But as a team, we can now try and help by… thinking back ‘this is what we've learned, in this team talk’…can we just try and use it? (P14)

Despite being largely positive about the training, and reporting an increased confidence in their ability to relate to patients, a majority of participants suggested that it would be difficult to identify the outcomes of the training.

Whatever impact, it's subtle and probably unseen, but probably there somewhere, but it's very hard to quantify it. (P9)

## 4 Discussion

This study aimed to explore staff perceptions of the utility of attachment theory for understanding patient behaviour within an IPCU. Thematic analysis led to the development of three themes that addressed the aims of the study: *engaging the reflective self*, *a new perspective on others* and *cohesive ward culture*.

The theme of *engaging the reflective self* adds to current literature by demonstrating the utility of attachment theory as a tool for facilitating self‐reflection in staff. Self‐reflection, guided by the attachment‐based cognitive behavioural models, provided staff with a chance to consider their own personal responsibility for patient behaviour. Whilst personal responsibility on behalf of staff has been identified as crucial for good inpatient care, feeling unable to provide optimal care has been linked to feelings of failure and moral distress in acute psychiatric staff (Jansen et al. 2020). In our study, attributing challenging behaviour to the patient's attachment‐related expectations, as opposed to a personal failure to provide adequate care on behalf of the staff member, facilitated a reduction in emotional reactivity within staff members. Decreased emotional reactivity, and a decreased sense of personal responsibility for patient behaviour, appears to have positively impacted staff psychological wellbeing.

The decreased emotional reactivity reported by staff may also help explain the shifts in staff perception that occurred at an interpersonal level. These shifts were captured under the theme *a new perspective on others*. Past research has found that negative perceptions of patient behaviour may be mediated by strong emotional responses, in particular worry, relating to the behaviour (Wheatley and Austin‐Payne 2009). A reduced level of emotional reactivity appeared to allow staff to access an increased capacity for reflection. Staff were more able to recognise that patient's challenging behaviour may be a manifestation of emotional regulation strategies, developed in response to early life experiences. This, in turn, was linked to greater empathy and more positive perceptions of patient behaviour. Adopting a more positive perspective of a patient's behaviour may have a self‐reinforcing effect. Staff who perceive their relationship with a patient as positive are less likely to view challenging behaviour as intentional (Berry et al. 2012).

Despite the apparent increase in reflective capacity following the training, staff offered limitations of the attachment‐focused approach. The ability to reflect on the relationship between early life experiences and patient behaviour was reported to be inhibited when faced with violence or clinical acuity. Participants evaluated the attachment‐focused approach as having little utility when assessing and managing risk. Future iterations of Safe & Secure may benefit from highlighting how the attachment‐focused approach can be used with the most unwell patients or in crisis situations.

Participants reported that being able to recognise and value individual differences in attachment styles across the team was helpful. Understanding the attachment needs of other team members is considered crucial to the development of a ‘well‐connected’ team (Bevington, Fuggle, and Fonagy 2015). Consistent with this, the high turnover of staff and the frequent reliance on temporary staff was cited as a barrier to the sustainability of the attachment‐focused approach. An awareness of attachment theory was also linked to a greater understanding of the behaviour of other staff members during stressful periods. Past qualitative research has shown that staff can experience regret and shame due to their actions, or lack of ability to act, during particularly distressing ward events such as physical restraint (Mooney and Kanyeredzi 2021). Self‐disclosure on behalf of the training facilitators appears to have modelled an absence of shame regarding personal attachment insecurity and had a beneficial effect on the implementation of the programme.

The third theme, *a cohesive ward culture*, was consistent with previous research in recognising the importance that staff placed on having a shared language with which to communicate about patients (Kramarz et al. 2023). The ability of team members to effectively share ideas and perspectives about patient care has been shown to strengthen team cohesion in mental health services (Short et al. 2019). Staff frequently commented on their use of attachment styles as a short‐hand way of advising colleagues about patients. Whilst framed as a positive development by participants, it can be recognised that factors other than attachment can influence patient behaviour and that the new shared language could lead to the misattribution of normal patient behaviour to their attachment style (Campbell, Allan, and Sims 2014).

Despite the changes in attitude that were captured across the three themes, almost all participants reported that it was difficult to identify the impact that Safe & Secure had on their clinical practice. Studies assessing team formulation in inpatient mental health wards report a similar effect. Staff recognise the indirect influence of psychology on their practice but struggle to identify examples of its direct application (McTiernan et al. 2021).

### 4.1 Limitations

The lead researcher (AD) was partially responsible for the delivery of the training programme as well as data collection and analysis, leading to a potential bias towards a favourable interpretation of how the data was analysed. In addition, the involvement of the ward Clinical Psychologist (DC) with the research may have influenced participant responses. Despite this, participants appeared comfortable in offering criticisms of Safe & Secure. The timeline of data collection must also be acknowledged. The final two participants to be interviewed had attended the 2‐h training session over 6 months earlier. These participants reported struggling to remember the content of the session in detail. It is possible that these participants were less reliable historians.

### 4.2 Recommendations for Future Research

Psychological interventions within acute mental health settings are associated with a reduction in patient incidents, improved staff perceptions of patients and improved relationships between staff and patients (Man, Wood, and Glover 2023). Measuring the impact of Safe & Secure on these variables, alongside rates of readmission, use of restraint, violence and aggression and length of stay, would provide further support for the use of the programme.

### 4.3 Conclusion

The current study provided a first opportunity to apply Newman‐Taylor et al.'s (2022) attachment‐based cognitive‐behavioural models within a clinical setting. These models have been suggested to improve care in acute settings by allowing staff to recognise, and positively influence, the cognitive, behavioural and affective processes common to patients who have attachment insecurity (Newman‐Taylor et al. 2022). Our findings were to some degree consistent with this hypothesis. In particular, the theme *a new perspective on others* recognised the power of the models in allowing staff greater insight into the attachment‐driven thoughts, behaviours and emotions of patients. As evidenced by the two other themes, staff also used the models to understand their own internal world and the internal world of their colleagues. Staff who were educated about attachment theory reported greater understanding and empathy for both patients and colleagues. Our study has provided evidence that attachment theory can provide a theoretical framework that can strengthen staff‐patient relationships within this setting. Staff from a range of disciplines were able to integrate attachment theory into their clinical practice within an 8‐week period. Newman‐Taylor et al.'s (2022) attachment‐focused cognitive behavioural model provides a suitable template for healthcare professionals seeking to develop an attachment‐focused inpatient service.

## 5 Relevance Statement

Developing strong relationships in acute psychiatric care is essential to help patients recover. This article helps mental health nurses to recognise the role of attachment in this setting. Hopefully, after engaging with this article, mental health nurses can further consider the role of attachment in their own work.

## Author Contributions

**Aiden Duffy:** conceptualisation, methodology, data collection, data analysis, project administration, writing – original draft, writing – review and editing. **Karen Goodall:** conceptualisation, methodology, supervision, validation, writing – review and editing. **David Carmichael:** conceptualisation, methodology, supervision, validation, writing – review and editing. **Sean Harper:** conceptualisation. **Katy Sivyer:** conceptualisation. **Kathy Carnelley:** conceptualisation. **Tess Maguire:** conceptualisation. **Katherine Newman‐Taylor:** conceptualisation, writing – review and editing.

## Conflicts of Interest

The authors declare no conflicts of interest.

## Data Availability

The data that support the findings of this study are available on request from the corresponding author. The data are not publicly available due to privacy or ethical restrictions.

## Appendix A Safe & Secure Training Overview

### A.1

Safe & Secure is an 8‐week staff training programme designed for staff working in inpatient settings, including those with no prior training in psychological approaches.

Learning objectives:

- To provide inpatient psychiatric care staff from across disciplines and bandings with an understanding of attachment theory
- To support inpatient psychiatric care staff in using their knowledge of attachment theory to identify patient attachment styles
- To support inpatient psychiatric care staff in offering attachment‐informed interventions tailored to the attachment needs of their patients

The programme begins with a 2‐h workshop hosted by two facilitators. Given their role in supporting psychological thinking and intervention in the inpatient setting, one of the facilitators should be the ward Clinical Psychologist. To support the credibility of the programme, and to be able to support the continued implementation of the training programme, it is preferable that facilitators are already well known to ward staff.

During the first hour of the workshop, participants are introduced, through didactic teaching alongside an accompanying PowerPoint presentation, to the following core concepts:

**Table:**

Time	Subject	Materials
0–5 min	The importance of relationships in mental health care	Slides
5–15 min	The origins of attachment theory	
15–25 min	Secure base, safe haven, secure attachment	
25–35 min	How do caregivers shape our attachment system?	
35–45 min	Attachment insecurity	
45–60 min	The relationship between attachment, trauma and complex mental health issues	

The second hour is a practical workshop during which the facilitators lead participants in applying attachment theory to their work on the ward:

**Table:**

Time	Focus	Materials
60–75 min	Newman‐Taylor et al.'s (2022) attachment‐based cognitive‐behavioural models are introduced. Case vignettes are used to demonstrate how the models can help to make sense of patient behaviour in the ward setting. Facilitators use the models to highlight useful strategies for meeting the attachment needs of individuals with different attachment representations	Attachment‐based CBT model templates, case vignettes
75–105 min	Staff discuss how these models apply to known patients on the ward. Staff work alongside the facilitators to formulate current inpatients and to consider how best to meet their attachment needs	
105–120 min	Facilitators recap the core ideas of the training session. Staff are then asked to continue to consider the role of attachment in driving patient behaviour as they return to work on the ward	Slides

The 2‐h workshop is followed by an 8‐week integration period. The integration period is managed by the ward Clinical Psychologist and centres upon the utilisation of the attachment‐based cognitive behavioural models to guide staff discussions within ward rounds, team formulations, reflective practice groups and case consultations:

**Table:**

Integration activity	Approach	Time, frequency
Ward round	Each patient's care is discussed by the MDT and care plans are updated as appropriate. Attachment is introduced as a relevant factor when adjusting care plans. The ward Clinical Psychologist takes a lead in prompting these discussions	3.5 h, one per week
Team formulation	A psychological formulation of patients' difficulties is co‐developed by the MDT, led by the ward Clinical Psychologist. Attachment is considered in the context of their early experiences and current behaviour	1.5 h, 3 weeks per month
Reflective practice groups	Staff are provided with a chance to reflect on the impact of their work on their own wellbeing. Attachment is offered as a useful model for understanding how staff respond to challenging situations	1.5 h, once per month
Case consultations	Staff ask the ward Clinical Psychologist for guidance with patient care. Staff are encouraged to consider how attachment is relevant to patients' behaviour	30 min, ad hoc

## References

[jpm13109-bib-0001] Baier, A. L. , A. C. Kline , and N. C. Feeny . 2020. “Therapeutic Alliance as a Mediator of Change: A Systematic Review and Evaluation of Research.” Clinical Psychology Review 82: 101921. 33069096 10.1016/j.cpr.2020.101921

[jpm13109-bib-0002] Berry, K. 2021. “Patient Social Functioning in Acute Mental Health Inpatient Wards: The Role of Emotional Regulation, Attachment Styles and Nurse‐Patient Relationships.” British Journal of Mental Health Nursing 10: 1–13.

[jpm13109-bib-0003] Berry, K. , L. Gregg , D. Vasconcelos e Sa , G. Haddock , and C. Barrowclough . 2012. “Staff–Patient Relationships and Outcomes in Schizophrenia: The Role of Staff Attributions.” Behaviour Research and Therapy 50, no. 3: 210–214. 22325807 10.1016/j.brat.2012.01.004

[jpm13109-bib-0004] Bevington, D. , P. Fuggle , and P. Fonagy . 2015. “Applying Attachment Theory to Effective Practice With Hard‐to‐Reach Youth: The AMBIT Approach.” Attachment & Human Development 17, no. 2: 157–174. 25782529 10.1080/14616734.2015.1006385

[jpm13109-bib-0005] Bolsinger, J. , M. Jaeger , P. Hoff , and A. Theodoridou . 2020. “Challenges and Opportunities in Building and Maintaining a Good Therapeutic Relationship in Acute Psychiatric Settings: A Narrative Review.” Frontiers in Psychiatry 10: 499994. 10.3389/fpsyt.2019.00965 PMC6974619 32009995

[jpm13109-bib-0006] Boniwell, N. , L. Etheridge , R. Bagshaw , J. Sullivan , and A. Watt . 2015. “Mental Health Nurses' Perceptions of Attachment Style as a Construct in a Medium Secure Hospital: A Thematic Analysis.” Journal of Mental Health Training, Education and Practice 10, no. 4: 218–233.

[jpm13109-bib-0007] Bowlby, J. 1969. Attachment and Loss: Vol. 1: Loss. New York, NY: Basic Books.

[jpm13109-bib-0008] Braun, V. , and V. Clarke . 2006. “Using Thematic Analysis in Psychology.” Qualitative Research in Psychology 3, no. 2: 77–101.

[jpm13109-bib-0009] Braun, V. , and V. Clarke . 2023. “Is Thematic Analysis Used Well in Health Psychology? A Critical Review of Published Research, With Recommendations for Quality Practice and Reporting.” Health Psychology Review 17: 1–24. 36656762 10.1080/17437199.2022.2161594

[jpm13109-bib-0010] Brennan, K. A. , C. L. Clark , and P. R. Shaver . 1998. “Self‐Report Measurement of Adult Attachment: An Integrative Overview.” In Attachment Theory and Close Relationships, edited by J. A. Simpson and W. S. Rholes , 46–76. New York: Guilford Press.

[jpm13109-bib-0011] Bretherton, I. 2013. “Internal Working Models of Attachment Relationships as Related to Resilient Coping.” In Development and Vulnerability in Close Relationships, edited by G. G. Noam and K. W. Fischer , 3–27. New York: Psychology Press.

[jpm13109-bib-0012] Bretherton, I. , and K. Munholland . 2018. “The Internal Working Model Construct in Light of Contemporary Neuroimaging Research.” In Handbook of Attachment: Theory, Research, and Clinical Applications, edited by J. Cassidy and P. R. Shaver , 3rd ed., 63–88. New York: Guilford Press.

[jpm13109-bib-0013] Bucci, S. , K. Berry , A. N. Danquah , and L. Johnstone . 2019. “How Can Attachment Theory Inform the Design and Delivery of Mental Health Services?” In Attachment Theory and Psychosis, edited by K. Berry , S. Bucci , and A. N. Danquah , 237–252. Oxon, UK: Routledge.

[jpm13109-bib-0014] Bucci, S. , N. H. Roberts , A. N. Danquah , and K. Berry . 2015. “Using Attachment Theory to Inform the Design and Delivery of Mental Health Services: A Systematic Review of the Literature.” Psychology and Psychotherapy: Theory, Research and Practice 88, no. 1: 1–20. 10.1111/papt.12029 24729543

[jpm13109-bib-0015] Campbell, R. , S. Allan , and P. Sims . 2014. “Service Attachment: The Relative Contributions of Ward Climate Perceptions and Attachment Anxiety and Avoidance in Male Inpatients With Psychosis.” Criminal Behaviour and Mental Health 24, no. 1: 49–59. 24014501 10.1002/cbm.1880

[jpm13109-bib-0016] Carr, S. C. , A. Hardy , and M. Fornells‐Ambrojo . 2018. “Relationship Between Attachment Style and Symptom Severity Across the Psychosis Spectrum: A Meta‐Analysis.” Clinical Psychology Review 59: 145–158. 29229220 10.1016/j.cpr.2017.12.001

[jpm13109-bib-0017] Clark, G. I. , A. J. Rock , L. H. Clark , and K. Murray‐Lyon . 2020. “Adult Attachment, Worry and Reassurance Seeking: Investigating the Role of Intolerance of Uncertainty.” Clinical Psychologist 24, no. 3: 294–305.

[jpm13109-bib-0018] De Ruysscher, C. , S. Vandevelde , P. Tomlinson , and S. Vanheule . 2020. “A Qualitative Exploration of Service Users' and Staff Members' Perspectives on the Roles of Inpatient Settings in Mental Health Recovery.” International Journal of Mental Health Systems 14: 1–13. 32165919 10.1186/s13033-020-00347-w PMC7060519

[jpm13109-bib-0019] Gillath, O. , and G. Karantzas . 2019. “Attachment Security Priming: A Systematic Review.” Current Opinion in Psychology 25: 86–95. 29621693 10.1016/j.copsyc.2018.03.001

[jpm13109-bib-0020] Herstell, S. , L. T. Betz , N. Penzel , et al. 2021. “Insecure Attachment as a Transdiagnostic Risk Factor for Major Psychiatric Conditions: A Meta‐Analysis in Bipolar Disorder, Depression and Schizophrenia Spectrum Disorder.” Journal of Psychiatric Research 144: 190–201. 34678669 10.1016/j.jpsychires.2021.10.002

[jpm13109-bib-0021] Hopkins, J. E. , S. J. Loeb , and D. M. Fick . 2009. “Beyond Satisfaction, What Service Users Expect of Inpatient Mental Health Care: A Literature Review.” Journal of Psychiatric and Mental Health Nursing 16, no. 10: 927–937. 19930367 10.1111/j.1365-2850.2009.01501.x

[jpm13109-bib-0022] Jansen, T. L. , M. H. Hem , L. J. Dambolt , and I. Hanssen . 2020. “Moral Distress in Acute Psychiatric Nursing: Multifaceted Dilemmas and Demands.” Nursing Ethics 27, no. 5: 1315–1326. 31631779 10.1177/0969733019877526

[jpm13109-bib-0023] Kobak, R. , and G. Bosmans . 2019. “Attachment and Psychopathology: A Dynamic Model of the Insecure Cycle.” Current Opinion in Psychology 25: 76–80. 29614483 10.1016/j.copsyc.2018.02.018 PMC6138578

[jpm13109-bib-0024] Kramarz, E. , C. L. M. Mok , M. Westhead , and S. Riches . 2023. “Staff Experience of Team Case Formulation to Address Challenging Behaviour on Acute Psychiatric Wards: A Mixed‐Methods Study.” Journal of Mental Health 32, no. 2: 412–423. 35037548 10.1080/09638237.2021.2022611

[jpm13109-bib-0025] Man, H. , L. Wood , and N. Glover . 2023. “A Systematic Review and Narrative Synthesis of Indirect Psychological Intervention in Acute Mental Health Inpatient Settings.” Clinical Psychology & Psychotherapy 30, no. 1: 24–37. 35997039 10.1002/cpp.2780 PMC10087275

[jpm13109-bib-0026] McGonagle, G. , S. Bucci , F. Varese , J. Raphael , and K. Berry . 2021. “Is Adult Attachment Associated With Engagement With Services? A Systematic Literature Review.” Journal of Mental Health 30, no. 5: 607–618. 31084388 10.1080/09638237.2019.1608922

[jpm13109-bib-0027] McTiernan, K. , L. Jackman , L. Robinson , and M. Thomas . 2021. “A Thematic Analysis of the Multidisciplinary Team Understanding of the 5P Team Formulation Model and Its Evaluation on a Psychosis Rehabilitation Unit.” Community Mental Health Journal 57: 579–588. 32737674 10.1007/s10597-020-00684-7

[jpm13109-bib-0028] Mikulincer, M. , and P. R. Shaver . 2019. “Attachment Orientations and Emotion Regulation.” Current Opinion in Psychology 25: 6–10. 29494853 10.1016/j.copsyc.2018.02.006

[jpm13109-bib-0029] Mooney, M. , and A. Kanyeredzi . 2021. “‘You Get This Conflict Between You as a Person and You in Your Role… That Changes You’: A Thematic Analysis of How Inpatient Psychiatric Healthcare Staff in the UK Experience Restraint, Seclusion, and Other Restrictive Practices.” International Journal of Mental Health Nursing 30, no. 6: 1703–1712. 34494346 10.1111/inm.12926

[jpm13109-bib-0030] Newman‐Taylor, K. , S. Harper , T. Maguire , K. Sivyer , C. Sapachlari , and K. B. Carnelley . 2022. “Attachment‐Based CBT Models for Psychosis: A PPI‐Informed Approach for Acute Care Settings.” Cognitive Behaviour Therapist 15: e55.

[jpm13109-bib-0031] Pipkin, A. , L. Hogg , and S. Armitage . 2021. “‘Someone on My Level’: A Meta‐Ethnographic Review of Therapeutic Relationships in Cognitive Behavioural Therapy for Psychosis.” Clinical Psychology & Psychotherapy 28, no. 5: 1297–1313. 33605515 10.1002/cpp.2578

[jpm13109-bib-0032] Short, V. , J. A. Covey , L. A. Webster , et al. 2019. “Considering the Team in Team Formulation: A Systematic Review.” Mental Health Review Journal 24, no. 1: 11–29.

[jpm13109-bib-0033] Staniszewska, S. , C. Mockford , G. Chadburn , et al. 2019. “Experiences of In‐Patient Mental Health Services: Systematic Review.” British Journal of Psychiatry 214, no. 6: 329–338. 10.1192/bjp.2019.22 30894243

[jpm13109-bib-0034] Wainwright, L. D. , K. Berry , C. Dunster‐Page , and G. Haddock . 2021. “Patient Social Functioning in Acute Mental Health Inpatient Wards: The Role of Emotional Regulation, Attachment Styles and Nurse–Patient Relationships.” British Journal of Mental Health Nursing 10, no. 4: 1–13.

[jpm13109-bib-0035] Walsh, J. , and J. Boyle . 2009. “Improving Acute Psychiatric Hospital Services According to Inpatient Experiences. A User‐Led Piece of Research as a Means to Empowerment.” Issues in Mental Health Nursing 30, no. 1: 31–38. 19148819 10.1080/01612840802500733

[jpm13109-bib-0036] Wheatley, M. , and H. Austin‐Payne . 2009. “Nursing Staff Knowledge and Attitudes Towards Deliberate Self‐Harm in Adults and Adolescents in an Inpatient Setting.” Behavioural and Cognitive Psychotherapy 37, no. 3: 293–309. 19393121 10.1017/S1352465809005268

[jpm13109-bib-0037] Wood, L. , C. Williams , J. Billings , and S. Johnson . 2019. “The Therapeutic Needs of Psychiatric In‐Patients With Psychosis: A Qualitative Exploration of Patient and Staff Perspectives.” BJPsych Open 5, no. 3: e45. 31530314 10.1192/bjo.2019.33 PMC6537445

